# Supplementary material for: Microfluidic Device for Droplet Pairing by Combining Droplet Railing and Floating Trap Arrays
Source: Micromachines (Basel). 2021 Sep 6;12(9):1076. doi: 10.3390/mi12091076 (PMC8470175; doi:10.3390/mi12091076)
Supplement: Supplementary file 1 [file micromachines-12-01076-s001.zip › micromachines-1349350 supplementary materials.pdf]

Microfluidic Device for Droplet Pairing by Combining Droplet Railing and Floating Trap Arrays

Margaux Duchamp, Marion Arnaud, Sara Bobisse, George Coukos, Alexandre Harari and Philippe Renaud

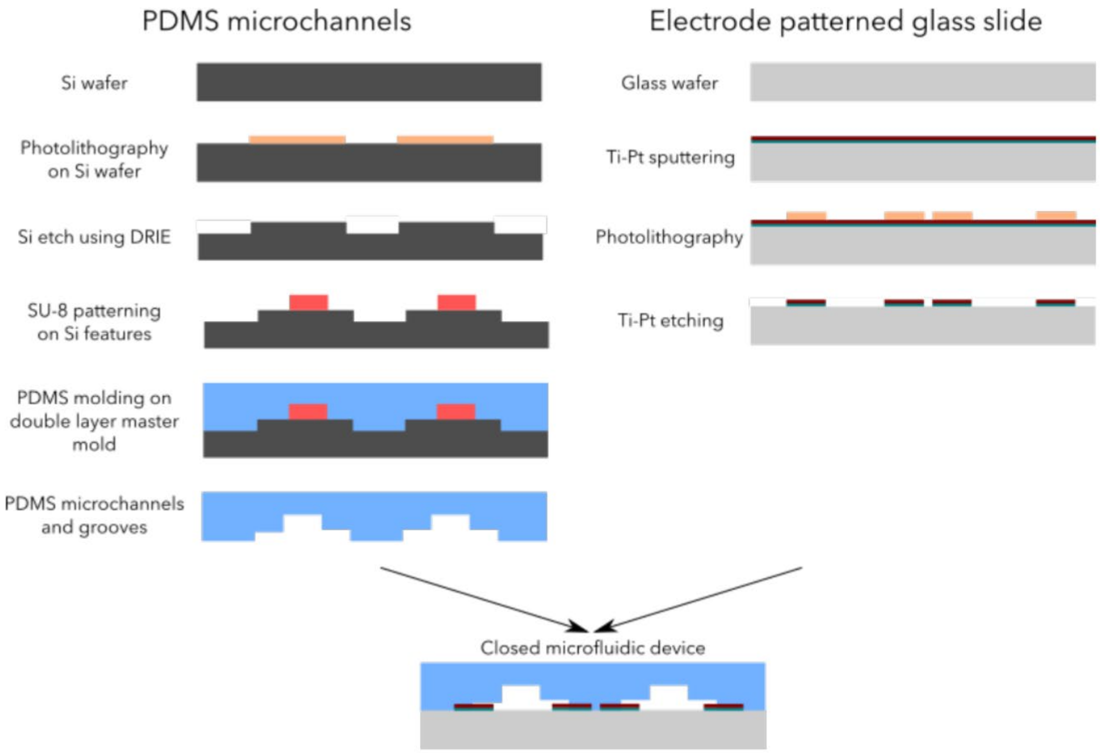

Figure S1. Simplified schematic of the process flow used to fabricate the microfluidic device. On the.left the fabrication steps to produce the PDMS microchannels. On the right the fabrication steps producing the Ti-Pt electrodes on glass. The final device is obtained by aligning and bonding the PDMS microchannels with the electrode patterned glass slide.

Video S1: Video of small droplets (94pL) continuing their way on the narrow rail at the droplet filtering region. The narrow rail is 10 μm wide while the larger rail is 20 μm wide. Both rails are 40 μm deep and are spaced by a gap of 10 μm. The images were acquired and are displayed at 14 FPS (real speed).

Video S2: Video of small droplets (94pL) being trapped in the smaller compartment of the trap. The images were acquired and are displayed at 14 FPS (real speed). The small compartment of the trap has a diameter of 40 μm.

Video S3: Video of big droplets (104pL) being diverted to the wider rail at the droplet filtering region and then being trapped in the wider compartment of the trap. The narrow rail is 10 μm wide while the larger rail is 20 μm wide. Both rails are 40 μm deep and are spaced by a gap of 10 μm. The images were acquired and

are displayed at 14 FPS (real speed). The big compartment of the trap has a diameter of 70 μm.

Video S4: Electro-coalescence (400V at 1kHz) of two droplets confined in the trap containing different aqueous phases. Images were acquired and are displayed at 200FPS (real speed).
